# Study protocol: a randomized controlled clinical trial on the use of electroacupuncture for postoperative abdominal distension following laparoscopic cholecystectomy

**DOI:** 10.3389/fsurg.2025.1557431

**Published:** 2025-08-21

**Authors:** Qi Kong, Wei Wang, Xin-Yi Chen, Li-Ming Chen, Long-Kai Sun, Shao-Hua Qu, Wen-Tao Wu, Jia-Hua Yang, Yu Shu, Sen Li, Pei-Hao Yin, Wei Li

**Affiliations:** ^1^ Department of General Surgery, Putuo Hospital, Shanghai University of Traditional Chinese Medicine, Shanghai, China; ^2^ Interventional Cancer Institute of Chinese Integrative Medicine, Shanghai University of Traditional Chinese Medicine, Shanghai, China; ^3^ Putuo People's Hospital, School of Medicine, Tongji University, Shanghai, China; ^4^ Department of Rehabilitation, Putuo Hospital, Shanghai University of Traditional Chinese Medicine, Shanghai, China; ^5^ Department of Rheumatology and Immunology, Shanghai TCM-Integrated Hospital, Shanghai University of Traditional Chinese Medicine, Shanghai, China; ^6^ Shanghai Academy of International Standardization for Traditional Chinese Medicine, Shanghai University of Traditional Chinese Medicine, Shanghai, China

**Keywords:** electroacupuncture, laparoscopic cholecystectomy, abdominal distension, enhanced recovery after surgery, clinical trial

## Abstract

**Background:**

In recent years, global cholecyst-related disorders have been increasing daily. Laparoscopic cholecystectomy (LC) is an advanced gallbladder surgical technique. However, pneumoperitoneum and various factors leading to abdominal distension and other gastrointestinal dysfunctions are common postoperative complications. Although postoperative gastrointestinal dysfunction can be treated with Avimopan, this medication also has adverse reactions such as nausea and vomiting. Acupuncture is one of the distinct treatment methods in TCM (Traditional Chinese Medicine), characterized by its simplicity, practicability, and minimal side effects. Previous studies have confirmed the effectiveness of acupuncture intervention for gastrointestinal functionality.

**Method:**

This study is a randomized, single-blind, controlled pilot clinical trial. We divided the patients into two groups: Electroacupuncture (EA) meridian acupoint group and EA non-meridian non-acupoint group. During the study period, both groups will receive routine care and education with an anticipated total of 36 individuals in each group. Both groups of patients will be treated with electroacupuncture (EA) after surgery, and in the morning of the day following the operation, with each session lasting 30 min. Specifically, the EA meridian acupoint group had intervention at the bilateral Zusanli (ST36) and Neiguan (PC6) points, while the EA non-meridian non-acupoints group had selected acupoint interventions at about 1.5 cm from ST36 and PC6. We will use the VAS (Visual Analogue Scale) and abdominal distension grading to evaluate the degree of patients’ postoperative abdominal distension 1–6 h and 24 h after surgery to obtain outcome assessments.

**Expected outcome:**

This study aims to scientifically and standardly evaluate the impact of EA in lessening post-LC abdominal distension through a clinical trial. We hope to get direct clinical evidence to demonstrate the role that EA can play in the ERAS (enhanced recovery after surgery) in patients post-LC. We hypothesise that the therapeutic effect of EA at meridian acupoints is superior to EA at non-meridian non-acupoint in the comprehensive intervention after LC.

**Clinical Trial Registration:**

http://www.chictr.org.cn, identifier ChiCTR2300073134.

## 1 Background

Gallbladder-related diseases are increasing worldwide in recent years (1). In the context of the high prevalence of gallbladder diseases, the application of laparoscopic cholecystectomy (LC) has significantly increased. LC is an advanced surgical technique for gallbladder removal that utilizes laparoscopic technology to perform the surgery through small abdominal incisions. It offers advantages such as minimal invasiveness, low complications, and fast postoperative recovery compared to traditional open surgery (2). Therefore, LC has become the preferred treatment option for common biliary diseases (3). During the LC procedure, the surgeon creates small incisions in the patient's abdominal wall and inserts laparoscopic instruments into the abdominal cavity to perform the surgery. The gallbladder and surrounding organs are visualized using a microscopic camera, and then the gallbladder is removed using the instruments. This surgical approach is mainly suitable for patients with gallstones, cholecystitis, and other related conditions, with a typical surgery duration of 1–2 h. Patients can quickly resume normal diet and activities postoperatively (4), although mild to moderate pain may be present. However, serious complications of LC may include damage to the common bile duct or other bile ducts (4), highlighting the significance of considering the potential complications associated with LC (5).

During the LC procedure, nitrogen or carbon dioxide is injected into the abdominal cavity for visualization of the internal organs (6). These gases need to be expelled from the body after the surgery, but sometimes they may remain in the abdominal cavity. As a result, abdominal distension and other gastrointestinal dysfunctions caused by pneumoperitoneum and various factors are common complications after LC (7, 8). According to an early study (9), 80% of patients with gallstones who underwent cholecystectomy experienced severe digestive disorders, suggesting that gallbladder inflammation may be a contributing factor to gastric bloating-related digestive disorders. Additionally, due to pneumoperitoneum, surgical trauma, and the effects of anesthesia, normal bowel movements often fail to recover within 24 h after the surgery. Furthermore, severe abdominal distension may impede venous blood flow in the inferior vena cava, leading to complications such as deep vein thrombosis (10). Therefore, improving abdominal distension, pain, and other gastrointestinal dysfunction after LC surgery plays an important role in promoting patient recovery and improving postoperative quality of life.

Postoperative gastrointestinal dysfunction can be treated with alvimopan, but this medication also has adverse effects such as nausea and vomiting (11), which makes it difficult to guarantee patient benefits. Acupuncture, a treatment used in Traditional Chinese Medicine (TCM) for centuries, is believed to have certain effectiveness in improving gastrointestinal function (12). For example, acupuncture is thought to treat irritable bowel syndrome through mechanisms such as gastrointestinal motility, visceral hypersensitivity, the immune system, neurotransmitters, and the brain-gut axis (13). Studies and our previous review have shown that acupuncture has gained attention as an effective method for treating postoperative gastrointestinal symptoms and intestinal motility disorders (14–17). Electroacupuncture (EA) after laparoscopic cholecystectomy (LC) significantly promotes postoperative intestinal function recovery in patients (18). It is currently unclear whether EA after LC can help improve gastrointestinal symptoms such as bloating. Furthermore, we aim to confirm the effectiveness of commonly used acupoints in restoring gastrointestinal function. Therefore, this study aims to evaluate the efficacy of EA in reducing postoperative bloating and promoting gastrointestinal recovery time after LC. We hypothesize that EA can further accelerate the recovery of intestinal function after LC.

## 2 Methods/design

### 2.1 Design

This study is a single-center, randomized controlled clinical trial. The study aims to evaluate the clinical efficacy and efficiency of EA in treating post-laparoscopic cholecystectomy bloating. This study is designed as a superiority trial, hypothesizing that the efficacy of EA at meridian acupoints is superior to sham EA in the integrative medical care after laparoscopic cholecystectomy. The protocol of this clinical trail is based on or refers to the following standard items: the Standard Protocol Items: Recommendations for Interventional Trials (SPIRIT) checklist (19) and its TCM extension (20); Consolidated Standards of Reporting Trials (CONSORT) (21); Standards for Reporting Interventions in Clinical Trials of Acupuncture (STRICTA) (22); WHO standard acupuncture point locations in the Western Pacific region. This clinical trial protocol is approved by the Ethics Committee of Putuo District Central Hospital, Shanghai (approval number: PTEC-A-2023-29-1) and registered with the Chinese Clinical Trial Registry (Registration number: ChiCTR2300073134).

### 2.2 Patient recruitment

**Figure 1:**
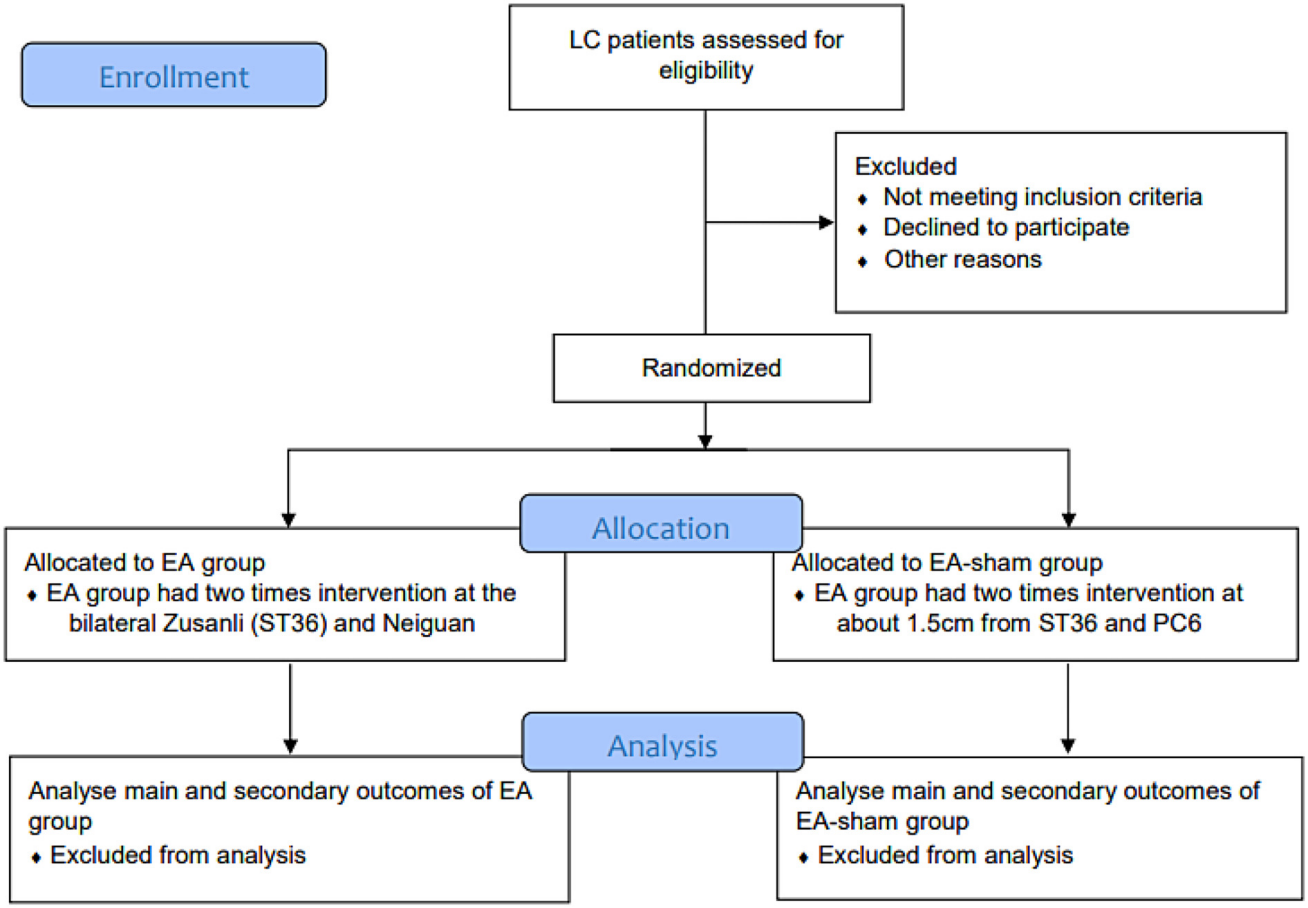
Flow chart.

This study is expected to include patients who undergo LC at the Department of General Surgery, Putuo Hospital, Shanghai University of Traditional Chinese Medicine, from July 1, 2023 to December 31, 2024. Patients who express interest will be recruited through preoperative interviews and pre-screening. After confirmation by clinical physicians and signing an informed consent form, baseline data will be collected. The criteria for pre-screening were defined as Patients with benign gallbladder diseases requiring LC as outlined in the “Expert Consensus on Surgical Treatment of Benign Gallbladder Diseases in China (2021)” (23) (Figure 1).

### 2.3 Patient selection criteria

#### 2.3.1 Inclusive criteria

1. Aged 18–70, both males and females will be included;
2. Diagnosed with benign gallbladder disease, in accordance with the indications for laparoscopic cholecystectomy as outlined in “Routine Laparoscopic Cholecystectomy” (24), requiring LC resection;
3. Signed informed consent form;
4. Possessing a physical status of American Society of Anesthesiologists (ASA) classification III or better, including the following three grades: Grade I: Healthy, well-nourished, and with normal organ function; Grade II: Mild systemic disease, with adequate compensatory mechanisms; Grade III: Severe systemic disease that limits activity but is not incapacitating;
5. Undergoing abdominal surgery for the first time. Voluntarily participating in this clinical study.

Only those who meet all the above criteria can be included.

#### 2.3.2 Exclusion criteria

1. Patients requiring simultaneous combined procedures;
2. Patients experiencing severe intraoperative or postoperative complications requiring intensive care;
3. Patients using medications affecting the intestines (e.g., Chinese herbal medicine) within one month;
4. Patients who have undergone acupuncture treatment within six months;
5. Patients with cardiac pacemakers;
6. Patients currently participating in other clinical studies within six months;
7. Patients with a history of syncope, epilepsy, or psychiatric disorders;
8. Patients undergoing epidural anesthesia;

Meeting any of the above criteria will result in exclusion.

#### 2.3.3 Eliminate and detachment criteria

Patients meeting the following criteria during the study will be eliminated:

1. Those found to not meet the above diagnosis and inclusion criteria after randomization;
2. Patients were converted to open surgery intraoperatively or diagnosed with other disorders intraoperatively and postoperatively.
3. Patients with poor compliance who fail to receive acupuncture treatment as prescribed;
4. Patients experiencing severe adverse reactions, complications, and special physiological changes that make it inappropriate to continue the trial.

Subjects meeting the following criteria during the study will be considered as dropouts:

1. Those found to not meet the inclusion criteria or meet any of the exclusion criteria after randomization;
2. Patients requesting to withdraw from the study;
3. Patients experiencing intolerable adverse events/serious adverse events, where the investigator judges that the risk of continuing participation in the trial outweighs the benefits;
4. Patients who lost to follow-up.

### 2.4 Randomization, blinding, and grouping

The experiment was grouped using SPSS 26.0 (IBM, USA). A random number table was generated, with “0” representing the “EA group” and “1” representing the “EA-sham group.” The grouping results will be written on paper, placed in sealed and opaque envelopes, and kept by a dedicated person. A researcher who did not know the grouping results opened the envelopes in sequence according to the envelope numbers and assigned patients to the corresponding treatment groups, with no one allowed to make unauthorized changes. The study strictly adhered to the three-separation principle of blinding, which means that observation recording, treatment operations, and data analysis were conducted separately. Random grouping was the responsibility of a dedicated person, observation recording was completed by non-operators, treatment operations were carried out according to the envelope grouping, and data statistics and analysis were the responsibility of a dedicated person to ensure the authenticity of clinical records. Patients in both EA groups were unaware of their treatment group.

### 2.5 Intervention

#### 2.5.1 Basic intervention

The study was based on all patients receiving Routine Standard Postoperative Care (RSPC) according to the ERAS consensus and the Chinese Pathway Management Guidelines (2018).

#### 2.5.2 Intervention of EA group

Provide EA treatment on the basis of comprehensive intervention. According to the “WHO Standard acupuncture point locations in the Western Pacific Region”.

##### 2.5.2.1 Bilateral Zusanli (St36)

On the anterior aspect of the leg, on the line connecting ST35 and ST41, 3 B-cun inferior to ST35.

##### 2.5.2.2 Bilateral Neiguan (Pc6)

On the anterior side of the forearm, between the tendons of the palmaris longus and the flexor carpi radialis, 2 B-cun proximal to the palmar wrist crease.

##### 2.5.2.3 Procedure

All patients are placed in a supine position and the acupoints are routinely disinfected. The 40 mm disposable sterile acupuncture needle (Hwato, Suzhou, China) was used. The needle was directly punctured 0.5∼1 cun (about 15–30 mm). The acupuncture manipulation is reinforcing and reducing to DeQi. The patient may not be fully awakened at the time of the first acupuncture, so we defined DeQi as the sensation of blockage felt by the acupuncturist during the maneuver. After, use an electric wire to connect both sides of ST36, using a continuous wave type with a frequency of 2 Hz and an intensity of 2–3 mA. There were two times of EA interventions, with the first intervention starting after the surgery, and the second intervention lasting 30 min on the morning of the second postoperative day. When taking the needle, gently press with your hand and use a clean cotton pad to prevent bleeding.

#### 2.5.3 Intervention of EA-sham group

We defined EA-sham as electroacupuncture at non-meridian and non-acupoint locations. Consistent with the intervention in the EA meridian acupoint group mentioned above, acupoint selection intervention was performed by opening approximately 1.5 cm next to the ST36 and PC6.

### 2.6 Outcomes

#### 2.6.1 Main outcomes

The primary outcomes are postoperative abdominal distension grading and postoperative abdominal distension VAS score.

1. Postoperative abdominal distension grading: Postoperative abdominal distension is divided into four levels: Grade 0 (score 0): no sense of distension; Grade 1 (score 1): mild distension, slight increase in abdominal wall tension, does not affect rest and sleep; Grade 2 (score 2): moderate distension, high abdominal wall tension, affects rest and sleep; Grade 3 (score 3): severe distension, high abdominal wall tension, unable to rest and sleep. The degree of distension is observed and recorded every hour for 1–6 h and 24 h postoperatively.
2. Postoperative abdominal distension VAS score: Postoperative abdominal distension, pain, and nausea will be assessed using the VAS method, with the degree of distension observed and recorded every hour for 1–6 h and 24 h postoperatively.

#### 2.6.2 Secondary outcomes

Secondary outcomes include the time for postoperative abdominal distension to disappear, length of hospital stay, postoperative pain, nausea and vomiting, time to first independent walking, time to first postoperative flatus, time to first postoperative defecation, postoperative complications, and other qualitative indicators for patients.

1. Time for postoperative abdominal distension to disappear: Refers to the difference between the time for postoperative abdominal distension to disappear and the end of the surgery. Patients and their family members should be carefully informed to observe and report the time when the postoperative abdominal distension sensation disappears, and the details should be recorded in the Case Report Form (CRF).
2. Postoperative pain, nausea, and vomiting: Postoperative pain and nausea will be assessed using the Visual Analog Scale (VAS). The degree of pain and nausea will be observed and recorded every hour from 1 to 6 h postoperatively and at 24 h, and the frequency of vomiting will be recorded.
3. Time to first postoperative flatus: Refers to the difference between the time of the first postoperative flatus and the end of the surgery. Patients and their family members should be carefully informed to observe and report the time of the first postoperative flatus, and the details should be recorded in the CRF.
4. Time to first postoperative defecation: Refers to the difference between the time of the first postoperative defecation and the end of the surgery. Patients and their family members should be carefully informed to observe and report the time of the first postoperative defecation, and the details should be recorded in the CRF.
5. Length of hospital stay: The time from the end of the surgery to discharge.
6. Postoperative complications: Other postoperative complications will be recorded in the CRF.
7. Other qualitative indicators for patients.

#### 2.6.3 Safety evaluation

The occurrence of adverse events (AEs) will be monitored through patient reports and medical staff evaluations during the study process. Clearly, mild AEs caused by acupuncture will mainly be reported by patients (such as subcutaneous congestion, prolonged acupuncture pain, etc.). Needle extraction bleeding is extremely common in acupuncture operations and has a certain therapeutic effect. Therefore, using dry cotton ball finger pressure to quickly stop mild needle bleeding is not considered an AE. If a serious AE occurs that leads to hospitalization, disability, life-threatening or death, it will be reported to the Ethics Committee within 24 h.

### 2.7 Sample size estimation

The trial is primarily aimed at preliminary testing the feasibility of evaluating the efficacy of acupuncture in treating post-LC abdominal distension using our outcomes. We define this clinical trial as a pilot trial prior to a large sample trial. According to the “Regulations for the Administration of Drug Registration in China” the sample size for exploratory clinical studies should be at least 20–30 cases. Based on the availability of funds, we selected 30 patients, anticipated a 20% dropout rate, and determined a sample size of 72 patients. The results of this study will contribute to calculating the appropriate sample size for further randomized clinical trials.

### 2.8 Statistical analysis

All statistical analyses will be conducted according to the intention-to-treat (ITT) principle, which includes patients who have received at least one session of EA treatment. All paper-based data will be electronically entered and analyzed using SPSS 26.0 (per-protocol set for robustness analysis of the results). In the statistical description, continuous data will be presented as mean ± standard deviation (M ± SD) or median and interquartile range, while categorical data will be presented as frequencies and percentages. Demographic and other baseline data will be compared using ANOVA and chi-square tests. Kaplan–Meier analysis will be used for key times of primary outcomes and secondary endpoints, and group comparisons will be made using the Breslow (generalized Wilcoxon) test. Differences in the incidence rates of nausea, vomiting, and abdominal distension will be compared using chi-square tests or the Mann–Whitney U test. The occurrence rates and severity of compliance will be analyzed using chi-square tests or the Mann–Whitney U test at a significance level of *α* = 0.05. Missing data will be handled using last observation carried forward and multiple imputation.

## 3 Discussion

In recent years, the application of acupuncture in the perioperative period has attracted increasing attention, which is considered part of the enhanced recovery after surgery (ERAS) protocol (17). Acupuncture has great potential in promoting the recovery of human body functions and is currently used in the postoperative stage to promote recovery, alleviate postoperative pain, and prevent common postoperative discomfort such as nausea and vomiting (25). It is worth noting that LC is a relatively simple surgery. Although related discomforts after the surgery, such as abdominal distension and pain, can be managed with relevant medications, the adverse reactions of drugs commonly used for postoperative gastrointestinal dysfunction, such as alvimopan, significantly limit their benefits for patients (26). Therefore, in the context of prioritizing patient-centered care, it is important to select an auxiliary intervention with minimal harm and fewer side effects.

EA is an optional technique. However, it is important to note that the effect of EA on specific acupoints is a significant issue. Therefore, in this study, we selected the acupoints ST36 and PC6 for research. ST36 and PC6 are commonly used acupoints for improving postoperative gastrointestinal function, and studies have shown (27) that palonosetron combined with acupuncture at ST36 and PC6 has a good preventive and therapeutic effect on postoperative nausea and vomiting, and contributes to the recovery of postoperative gastrointestinal function in patients. Therefore, in this study, our aim is to understand whether EA at specific acupoints is truly effective and whether simple acupoint treatment (only two single acupoints) helps improve postoperative abdominal distension and other gastrointestinal dysfunctions and discomfort in patients undergoing LC. Although acupuncture has minimal side effects and is relatively safe, it also presents the issue of pain. The excessive use of acupoints may likely subject patients to more pain during the treatment process, which is also a significant part of the patient's subjective experience. Therefore, the effectiveness of two single acupoints may also provide a reference for simplified acupoint prescriptions. At the same time, we also hope to further confirm the effectiveness of meridian acupoints, thereby providing a foundation for further research.

### 3.1 Strengths and limitations of this study

(1) Acupuncture has great potential in promoting the recovery of human body functions and is currently used in the postoperative stage; (2) ST36 and PC6 are commonly used acupoints for improving postoperative gastrointestinal function; (3) The acupuncturists performing the intervention had over ten years of clinical acupuncture experience, which was considered difficult to blind.

## 4 Limitations

In this study, there are certain limitations. Firstly, it is a single-center study, which may limit the generalizability of the study results. Secondly, in our study, we were unable to blind the acupuncturist because the acupuncturists performing the intervention had over ten years of clinical acupuncture experience, which was considered difficult to blind (28), so we did not implement blinding of the acupuncturists.

## Data Availability

The original contributions presented in the study are included in the article/Supplementary Material, further inquiries can be directed to the corresponding authors.
